# Physiological and Cellular Responses Caused by RNAi- Mediated Suppression of Snf7 Orthologue in Western Corn Rootworm (*Diabrotica virgifera virgifera*) Larvae

**DOI:** 10.1371/journal.pone.0054270

**Published:** 2013-01-18

**Authors:** Parthasarathy Ramaseshadri, Gerrit Segers, Ronald Flannagan, Elizabeth Wiggins, William Clinton, Oliver Ilagan, Brian McNulty, Thomas Clark, Renata Bolognesi

**Affiliations:** Department of Biotechnology, Monsanto Company, Chesterfield, Missouri, United States of America; St. Georges University of London, United Kingdom

## Abstract

Ingestion of double stranded RNA (dsRNA) has been previously demonstrated to be effective in triggering RNA interference (RNAi) in western corn rootworm (WCR, *Diabrotica virgifera virgifera* LeConte), providing potential novel opportunities for insect pest control. The putative Snf7 homolog of WCR (DvSnf7) has previously been shown to be an effective RNAi target for insect control, as DvSnf7 RNAi leads to lethality of WCR larvae. Snf7 functions as a part of the ESCRT (Endosomal Sorting Complex Required for Transport) pathway which plays a crucial role in cellular housekeeping by internalization, transport, sorting and lysosomal degradation of transmembrane proteins. To understand the effects that lead to death of WCR larvae by DvSnf7 RNAi, we examined some of the distinct cellular processes associated with ESCRT functions such as de-ubiquitination of proteins and autophagy. Our data indicate that ubiquitinated proteins accumulate in DvSnf7 dsRNA-fed larval tissues and that the autophagy process seems to be impaired. These findings suggest that the malfunctioning of these cellular processes in both midgut and fat body tissues triggered by DvSnf7 RNAi were the main effects leading to the death of WCR. This study also illustrates that Snf7 is an essential gene in WCR and its functions are consistent with biological functions described for other eukaryotes.

## Introduction

Gene silencing by RNA interference (RNAi) has been used as a powerful tool for studying gene function in a variety of organisms. In the recent decade, RNAi has been gaining momentum as a biotechnological tool for insect control [1], [2], [3]. However, the success of this technology depends on the ability of insect species to be susceptible to ingested double-stranded RNA (dsRNA) at levels that result in commercial efficacy. Although RNAi experiments have been efficacious in a number of insect species [4], successful initiation of the RNAi pathway through feeding of dsRNA has only been documented in a few insect species [5], [6]. One of the insect species responsive to oral dsRNA is the western corn rootworm (WCR), *Diabrotica virgifera virgifera*, an economically important insect pest of corn. Baum et al [1] evaluated several candidate target genes in diet bioassays, and determined that genes encoding proteins with essential functions were found to cause lethality of WCR larvae at low dsRNA concentrations. One of the potential RNAi targets found in this study was a putative homolog of yeast Snf7.

Snf7 is a class E vacuolar sorting protein conserved in many organisms such as yeast (Vps32; [7]), humans (hSnf7 or CHMP4; [8]), mouse (mSnf7; [9]), fruit fly *Drosophila* (Shrub; [10]), nematode, *Caenorhabditis elegans* (CeVPS32.1 & CeVps32.2; [11]), and *Arabidopsis thaliana* (At2g19830 & At4g29160; [12]). Snf7 belongs to the ESCRT (Endosomal Sorting Complex Required for Transport)–III complex, which has been shown to be involved in sorting of transmembrane proteins en route to lysosomal degradation through the endosomal-autophagic pathway in multiple organisms [11], [13], [14], [15], [16]. The first step in the endosomal-autophagic pathway is endocytosis of membrane receptor proteins which are then delivered to early endosomes from where they are either recycled to the plasma membrane or sorted to the lysosome for degradation [17], [18], [19]. The second step is ubiquitination of the receptors, which aids in the process of specifying which proteins are targeted to be transported to the lysosome lumen for degradation in the endocytic pathway. The ubiquitinated cargo (receptor proteins) are recognized and sorted by the ESCRT pathway into the invaginations of the endosomal membranes that involves de-ubiquitination and recycling of ubiquitin [20]. Removal of ubiquitin occurs before the cargo enters the luminal vesicles of multi-vesicular bodies (MVB). Finally, MVB fuse with lysosomes to sequester the cargo through lysosomal degradation or autophagy [21]. ESCRT-III components play critical roles in distinct steps of this pathway [22], [23].

The ESCRT pathway is also a key regulator of biological processes important for eukaryotic cell growth and survival [24], [25], [26], [27]. The role of ESCRT proteins in endosomal sorting is well conserved from yeast to human and several studies have revealed additional functional roles in biological processes such as viral budding, cytokinesis and regulation of gene transcription [20], [22], [23], [28], [29]. Snf7 RNAi in mammalian and nematode systems revealed that Snf7 functions in multiple cellular processes [9], [11], [30]. However, despite Snf7 functional importance in these limited number of organisms, little is known about Snf7 presence and function in other organisms. Among insects, some of the functions of ESCRT-III components, including Vps32/Snf7, have been studied in detail only in *Drosophila* using mutant genetic screening analysis [16], [30].

We have demonstrated that feeding of dsRNA of a putative WCR Snf7 homolog (DvSnf7) to larvae caused severe stunting after five days of exposure followed by the death of the larvae. The insecticidal activity of DvSnf7 dsRNA occurred through the RNAi pathway leading to DvSnf7 suppression at mRNA and protein levels, and subsequent systemic spread of RNAi effect to other tissues distal to the midgut [31]. In order to gain a better understanding of the cellular processes that lead to mortality of WCR larvae after ingestion of Snf7 dsRNA, we investigated the functions of Snf7 by observing changes in the endosomal-autophagic pathway at cellular level after DvSnf7 dsRNA treatment of WCR larvae. The data gathered in this study suggest that the impairment of specific cellular processes lead to WCR death and that Snf7 functions are conserved in this coleopteran insect.

## Results and Discussion

Snf7 is present in most eukaryotic organisms. There are three human Snf7 homologs [8] and two mouse Snf7 homologs [9], of which, mSnf7-2 knockout mice died during embryogenesis [9]. Two Snf7 homologs have also been reported for *C. elegans* (CeVps32.1 & CeVps32.2) [32]. RNAi or deletion of CeVps32.1 caused lethality at multiple developmental stages of *C. elegens* [32] and feeding of bacteria expressing dsRNA of CeVps32.1 showed almost 100% mortality in *C. elegans* [11]. These studies demonstrated that Snf7 is a vital gene involved in eukaryotic developmental processes and different isoforms may have specific functions. However, a single Snf7 homolog (Shrub) was identified and characterized in *Drosophila*. The *shrub* mutants embryos died before reaching the larval stage [30]. We examined our WCR transcriptome/genomic database and found a single gene homologous to Snf7 (DvSnf7) (data not shown). In addition, Southern blot analysis of WCR genomic DNA confirmed the existence of single copy of DvSnf7 (Fig. S1).

**Figure 1:**
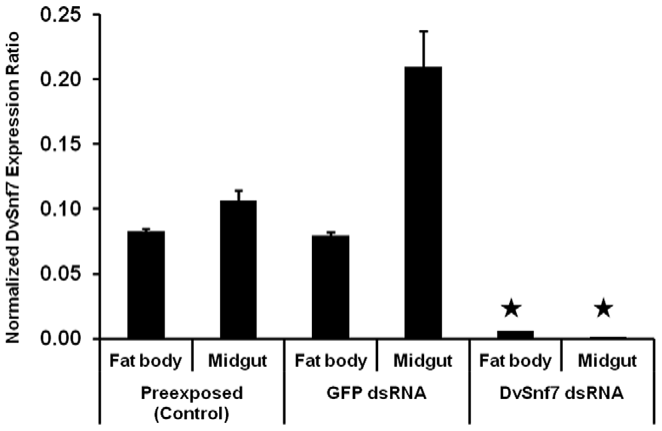
Suppression of DvSnf7 in WCR tissues as determined by real-time PCR. DsRNA for DvSnf7 and GFP (control) were overlaid on diet at 1 µg/ml of diet. Second instar WCR larvae were exposed to dsRNAs for five days. Midgut and fat bodies were dissected from WCR for total RNA extraction. Tissues from WCR larvae sampled prior to dsRNA exposure were used as the control. Mean ± S.E. of three biological replicates are shown. Stars represent values significantly different from controls (p = 0.050; t-test; SAS 9.2).

Feeding of DvSnf7 dsRNA to WCR larval stages results in stunting after five days of exposure followed shortly by lethality of larvae [31]. To investigate the mechanism of DvSnf7 RNAi leading to death, midgut (primary contact tissue with dsRNA via oral feeding) and fat bodies (primary metabolic tissue and nutrient sensor) tissues were analyzed after five days of exposure to DvSnf7 dsRNA. Suppression of DvSnf7 mRNA levels was confirmed by real-time PCR in midgut and fat body tissues of larvae that were fed on DvSnf7 dsRNA (Fig. 1). DvSnf7 mRNA levels were down-regulated by approximately 13- and 140-fold in fat bodies and midguts respectively, when compared to tissues from insects fed with green fluorescent protein (GFP) dsRNA or in tissues of pre-exposed insects (controls). These results indicate that the RNAi effect is systemic in WCR, which is consistent with previous data demonstrating the spread of RNAi beyond the midgut in WCR; suppression of DvSnf7 mRNA levels is observed in the carcass tissues by oral ingestion of DvSnf7 dsRNA [31] and by injection of DvvLaccase2 in WCR larvae [33]. Due to multiple sources of data indicating the presence of systemic RNAi in WCR, we analyzed the cellular effects of DvSnf7 RNAi in both midgut and fat body tissues.

**Figure 2:**
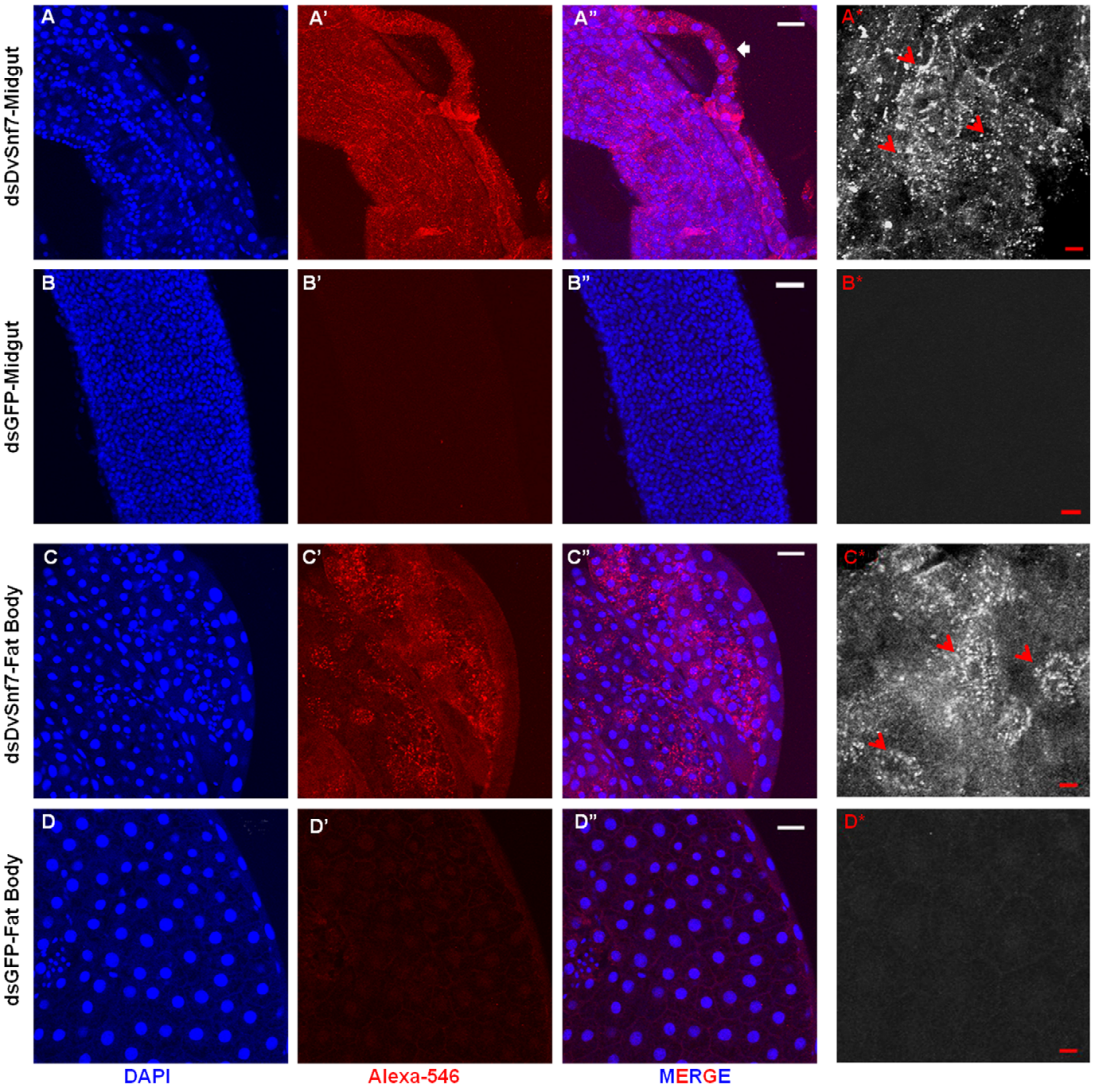
Immuno staining of WCR tissues with Anti-ubiquitin. DsRNA for DvSnf7 (dsDvSnf7) and GFP (dsGFP) were overlaid on diet at 1 µg/ml of diet. Second instar WCR larvae were exposed to dsRNAs for five days. Whole mounts of midgut and fat bodies were used for immuno-staining. Panels A, B, C, D show nuclear DNA (DAPI) staining of midgut and fat bodies. Panels A’, B’, C’, D’ show Alexa-546 detecting ubiquitin. Panels A”, B”, C”, D” show merged images of DAPI and Alexa-546. Panels A*, B*, C*, D* show monochrome images of respective treated tissues at higher magnification. Red arrowheads mark the ubiquitin puncta. Scale Bar: White –50 µm, Red –10 µm.

First, we mapped the endosomal protein sorting activity in the midguts and fat bodies of DvSnf7 and GFP dsRNA-fed larvae using an anti-ubiquitin antibody and immunohistochemistry (Fig. 2). Normally, the protein cargoes destined for lysosomal degradation are sorted by the ESCRT pathway via ubiquitination. Subsequent removal of ubiquitin from cargoes requires the enzymatic activity of the de-ubiquitinating enzyme Doa4 (degradation of alpha-2) and recruitment of Doa4 to the endosome requires correct assembly of ESCRT-III [34].

In our study, prominent intracellular ubiquitin staining was observed in the midguts of DvSnf7 dsRNA-fed insects (Fig.2; panel A’). The ubiquitin signals were visualized throughout the midgut and also in the adjoining malphigian tubules in the perinuclear regions (Fig. 2; panel A”). Ubiquitin puncta were distinct in the midgut epithelium in the DvSnf7 dsRNA-fed larvae at higher magnification (Fig. 2; panel A*). In contrast, no ubiquitin signal was detected in the midgut of the GFP dsRNA-fed control larvae representing absence of accumulated ubiquitinated protein, (Fig. 2; panels B series). Similarly, staining of fat bodies of DvSnf7 dsRNA-fed larvae with anti-ubiquitin antibody revealed prominent patterns of evenly dispersed ubiquitin puncta (Fig. 2, panel C’). Ubiquitin signals were significantly enhanced around the nuclei in the fat body cells (Fig. 2, panel C”, C*). These patterns were absent in fat body tissue of GFP dsRNA-fed larvae (Fig. 2, panel D”, D*). No fluorescent signals or puncta were observed in the midguts and fat bodies of either DvSnf7 or GFP dsRNA-fed larvae in the absence of primary anti-ubiquitin antibody (negative control; data not shown). These results indicate that DvSnf7 RNAi causes the accumulation of ubiquitinated proteins.

Loss of function of ESCRT-III subunits has been previously reported to cause accumulation of ubiquitinated proteins in multiple organisms [14], [16], [35], [36]. In *Drosophila,* all ESCRT mutants, including *vps32,* displayed severe intracellular ubiquitin accumulation in the mosaic eye discs [16]. Besides ESCRT proteins, suppression of Waharan, a protein that was shown to act upstream of ESCRT-III, *Shrub* in endosomal trafficking pathway, caused the appearance of prominent ubiquitinated puncta, reflecting the accumulation of proteins that fail to be stripped of their ubiquitin tags in the muscle tissue of *Drosophila* [37]. In the same study, it was shown that ubiquitin accumulation was a common feature upon interference with endocytic trafficking, as it was visualized in mutants of endosomal markers (*Rab5, Rab7*) and *Shrub* (a dominant-negative form of Snf7).

In line with these observations, suppression of DvSnf7 in WCR larval tissues leads to accumulation of ubiquitinated proteins indicating the requirement of this putative ESCRT-III component for protein sorting. Studies have demonstrated that de-ubiquitination carried out by Doa4 is recruited by Bro1, which interacts with ESCRT-III component Vps32, thereby connecting Doa4 and the ESCRT pathway [38], [39], [40]. Hence, it appears that suppression of DvSnf7 in WCR interferes with the de-ubiquitination process, leading to the accumulation of ubiquitinated proteins, impairment of protein sorting and degradation of proteins.

Second, we examined the autophagic response of WCR to DvSnf7 dsRNA treatments using the Lysotracker marker as previously described [41], [42]. Autophagy is the process through which the cellular proteins and other cytoplasmic components are cleared through lysosomal degradation [21]. The autophagic process can be stimulated in response to environmental signals such as nutritional deficit (starvation), which results in an autophagy-dependent expansion and acidification of the lysosomal compartments. Rusten et al. [43] demonstrated that several components of ESCRT-I, -II, and –III are required for autophagy in *Drosophila*. Insect fat bodies are specialized in mounting a particularly robust autophagic response to starvation, serving to provide a reserve source of nutrients for other critical tissues [44].

**Figure 3:**
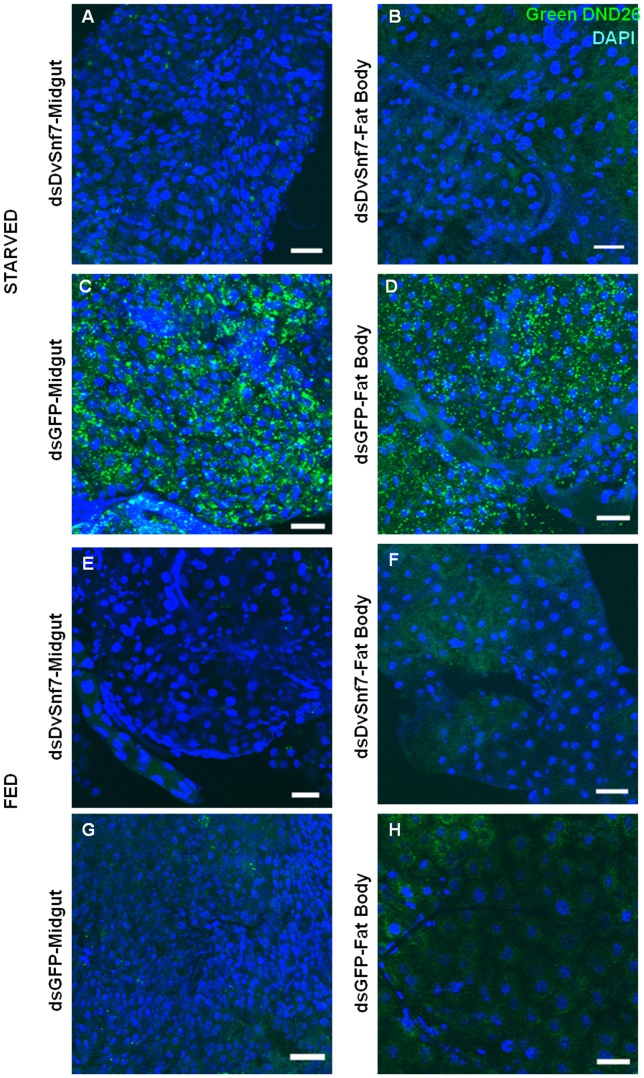
Lysotracker staining of WCR tissues. DsRNA for DvSnf7 (dsDvSnf7) and GFP (dsGFP) were overlaid on diet at 1 µg/ml of diet. One set- of second instar WCR larvae were exposed to dsRNAs for four days and then starved for 24 h. Another set of larvae were exposed to dsRNAs continuously for five days. Midgut and fat bodies were dissected from both sets of larvae and used for Lysotracker staining. Panels A–D show Lysotracker and nuclear staining (DAPI) merged images of starved samples. Panels E–H show Lysotracker and nuclear staining (DAPI) merged images of continuously fed samples. Green punctate marks denote Lysotracker staining that detects active acidic lysosomes undergoing autophagy (panels C &D). Scale Bar: 50 µm.

In our study, under starvation conditions, intense punctate staining indicative of acidic lysosome activity appeared in all cells of midgut and fat body tissues of dsGFP treated larvae (Fig. 3, C &D). However, starvation-induced formation of Lysotracker stain-positive structures was severely inhibited in both midguts and fat body tissues of DvSnf7 dsRNA-fed larvae (Fig. 3, A & B). Under normal dietary conditions, midguts and fat body tissues failed to incorporate Lysotracker and punctate fluorescent signals were observed in neither DvSnf7 nor GFP dsRNA-fed larvae (Fig. 3, E-H). No fluorescent signals or puncta were observed in the midguts and fat body tissues of either DvSnf7 or GFP dsRNA starved or fed larvae that were incubated in dye solution free of Lysotracker (negative control; data not shown). At higher magnification, Lysotracker puncta were observed mostly in the perinuclear region of the fat body and midgut cells of starved GFP RNAi insects (Fig. S2, A &B). No Lysotracker staining was observed in starved DvSnf7 RNAi tissues (Fig. S2, C& D); or in the tissues of GFP and DvSnf7 dsRNA fed insects (data not shown). The reduction of starvation-induced Lysotracker staining following suppression of DvSnf7 suggests that acidic lysosome activity is disrupted, with a possible effect on autophagy. These data indicate that suppression of DvSnf7 disrupts starvation-induced autophagy in both midgut and fat bodies of WCR.

Since autophagy is a lysosomal-mediated process of cytoplasmic degradation and starvation induces autophagy, we were able to differentiate the effects of DvSnf7 RNAi in this stimulated condition but not under the normal dietary conditions using Lysotracker staining. Similar observations have been made in *Drosophila*, where fat body tissue dissected from fed animals failed to incorporate Lysotracker (or occasionally displayed faint diffuse staining) while intense punctate Lysotracker staining was observed in the fat body of starved animals [42]. Our data indicating the inhibition of autophagic degradation in DvSnf7 dsRNA -fed larval tissues are in agreement with the study in *Drosophila*, where mutations in components of ESCRT-I (*Vps25*), -II (*Vps28*) or –III (*Vps32*) impacted starvation-induced Lysotracker staining in the larval fat body [45]. Mutations in ESCRT-III subunits have also been implicated in the inhibition of autophagic degradation in mammalian systems [14], [35]. In addition, *C. elegans* yolk proteins tagged with GFP showed increased fluorescence in the ESCRT-III (*Vps32.2*) mutant embryos [11]. However, in another isoform mutant, *Vps32.1*, these bright irregular puncta were not observed and the embryos showed 100% lethality. Kim et al. [11] predicted that these puncta in *Vps32.2* represented overlapped vescicles containing yolk proteins that failed to be further degraded, presumably by a block in their fusion with lysosomes. This study suggests the involvement of one of the isoforms of CeVps32 in endosomal to lysosomal protein degradation.

Malfunctioning of ESCRT-III specific functionalities such as de-ubiquitination and autophagy have been previously attributed to Snf7 depletion. However, studies in *Drosophila* eye discs suggest that Vps32/Snf7 may also be required for endocytosis of receptors, which is the initial step of the ESCRT pathway [16]. These authors demonstrated that *Vps32/Snf7* mutants displayed less endosomal accumulation of Notch and increased more plasma membrane localization than other ESCRT mutants. Other components of ESCRT complexes and vacuolar sorting proteins (Vps34) have also been shown to be involved in the proper functioning of endocytosis [11], [45], [46].

**Figure 4:**
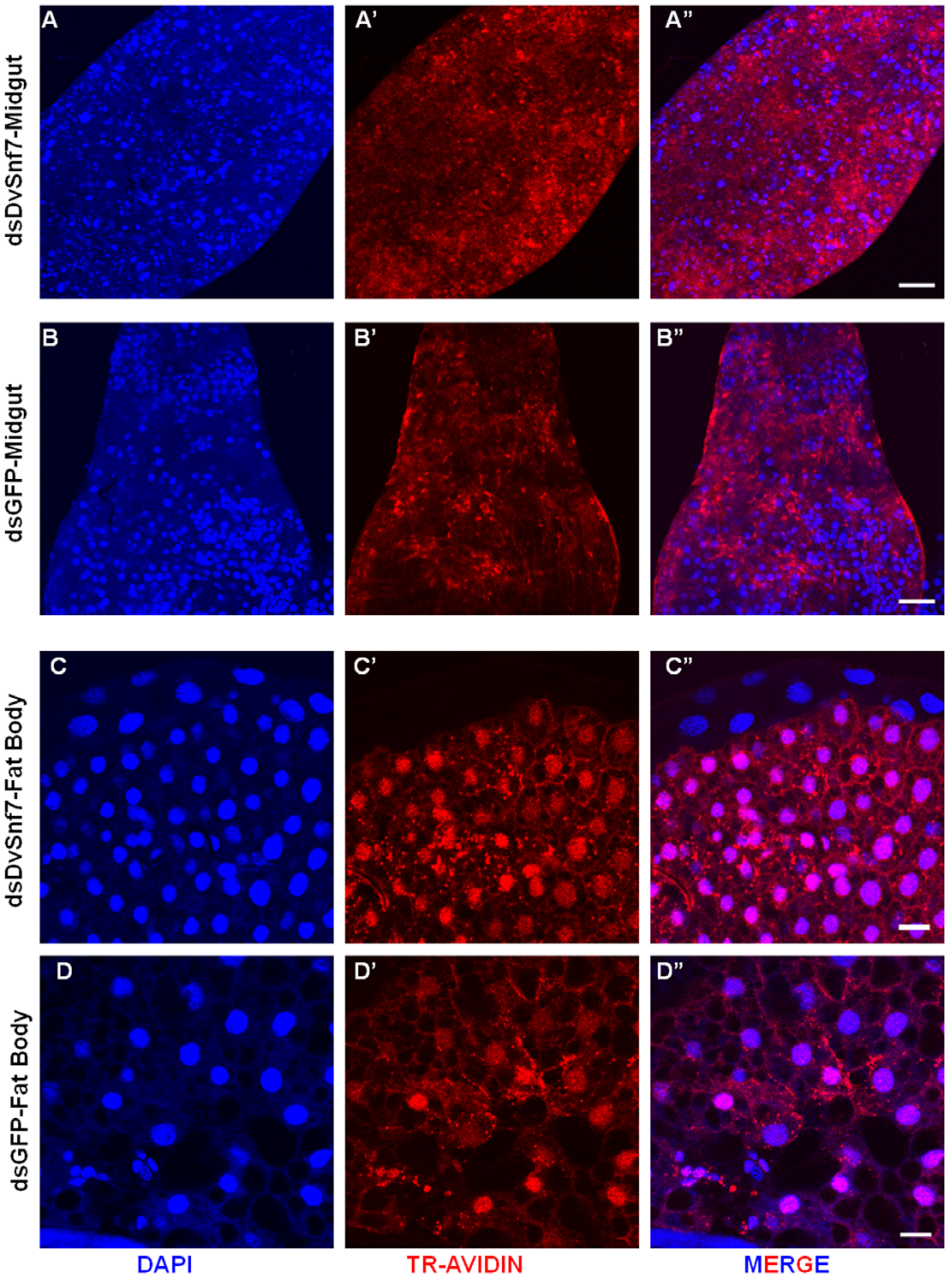
Avidin staining in WCR tissues. DsRNA for DvSnf7 (dsDvSnf7) and GFP (dsGFP) were overlaid on diet at 1 µg/ml of diet. Second instar WCR larvae were exposed to dsRNAs for five days. Midgut and fat bodies were dissected from WCR for staining. Panels A, B, C, D show nuclear DNA staining (DAPI) in midgut and fat bodies. Panels A’, B’, C’, D’ show Texas-Red conjugated avidin staining. Panels A”, B”, C”, D” show merged images of DAPI and Texas-Red. Scale Bar: A” & B”- 50 µm, C” & D” – 20 µm.

The endocytosis process involves internalization of extracellular materials and transmembrane proteins into the cell membrane for intracellular trafficking [11]. In this context, we monitored the effects of DvSnf7 RNAi on the endocytotic ability of WCR larval tissues by using the fluorescent endocytic tracer Texas-Red-conjugated Avidin (TR-Avidin). TR-Avidin has been successfully used to study the endocytosis process in *Drosophila* [47]. Midguts and fat bodies isolated from larvae after five days of exposure to either DvSnf7 or GFP dsRNAs were subjected to a pulse-chase treatment with TR-Avidin (Fig. 4). TR-Avidin was efficiently internalized by the midgut epithelial cells as well as fat body tissues of both DvSnf7 and GFP dsRNA-fed larvae (Fig. 4, panels A-D series). Cytoplasmic and some nuclear (co)-localization were observed in the midguts (Fig. 4; panels A” & B”). Distinct intracellular localization was observed in the fat body cells of both treatments with nuclear co-localization (Fig. 4; panels C” & D”). No fluorescent signals were observed when tissues were incubated in medium without TR-Avidin (negative control; data not shown). These results indicate that Avidin can be internalized by the cells, even with reduced levels of DvSnf7, indicating that DvSnf7 RNAi in WCR does not impair endocytosis, in general. Kim et al. [11] investigated the roles of *C. elegans* ESCRT components by monitoring endocytosis of a yolk protein fused to GFP in the oocytes of nematodes subjected to RNAi against each ESCRT component, including CeVps-32.2 (a Snf7 homolog). The pseudocoelomic accumulation of GFP tagged yolk proteins was interpreted as inefficient endocytosis. It was reported that suppression of some of the components of ESCRT-II (CeVps-25, CeVps-36) and ESCRT-III (CeVps-20, CeVps-24) hampered endocytosis but not CeVps32.2, which is consistent with our findings. Double RNAi treatments of CeVps-20, CeVps24, and CeVps-25 also showed increased accumulation of yolk proteins in the pseudocoelom of worms, indicating that CeESCRT-II and –III complexes may play roles in the uptake of yolk proteins [11]. However, accumulation of yolk proteins and MVB biogenesis were not compromised in *Drosophila Vps32* mutant oocytes [16].

Induction of apoptosis is one of the potential pathways that can lead to cell death and mortality. To investigate whether this is a potential mechanism for apoptosis in the mortality of WCR larvae, TUNEL staining was performed in midgut and fat body tissues of larvae fed with DvSnf7 and GFP dsRNAs after 5 days of exposure. TUNEL staining was negative in both midgut and fat body tissues of larvae fed with DvSnf7 dsRNA (Fig. S3; panels A & B). Similarly, TUNEL staining was absent in the tissues of larvae fed with GFP dsRNA (Fig. S3; panels C & D). A subset of the samples was treated with DNAse (positive control) and showed distinct TUNEL staining in the nuclei of both midguts and fat bodies of larvae fed with either DvSnf7 or GFP dsRNA (Fig. S3; panels E, F, G, H). The samples incubated with labeling solution lacking DNAse (negative control) did not show any TUNEL staining (data not shown). These results indicate that apoptosis is not the mechanism causing cell death of WCR larvae fed with DvSnf7 dsRNA. Similar results were observed in mouse cortical neurons infected with mSnf7-2 siRNA lentivirus. TUNEL staining was absent in these neurons indicating that apoptosis was not involved in the cell death and neural cell loss caused by ESCRT-III dysfunction [9]. This data indicate that apoptosis is not the primary cause of larval cell death and that ESCRT-III related functionalities might be involved.

**Figure 5:**
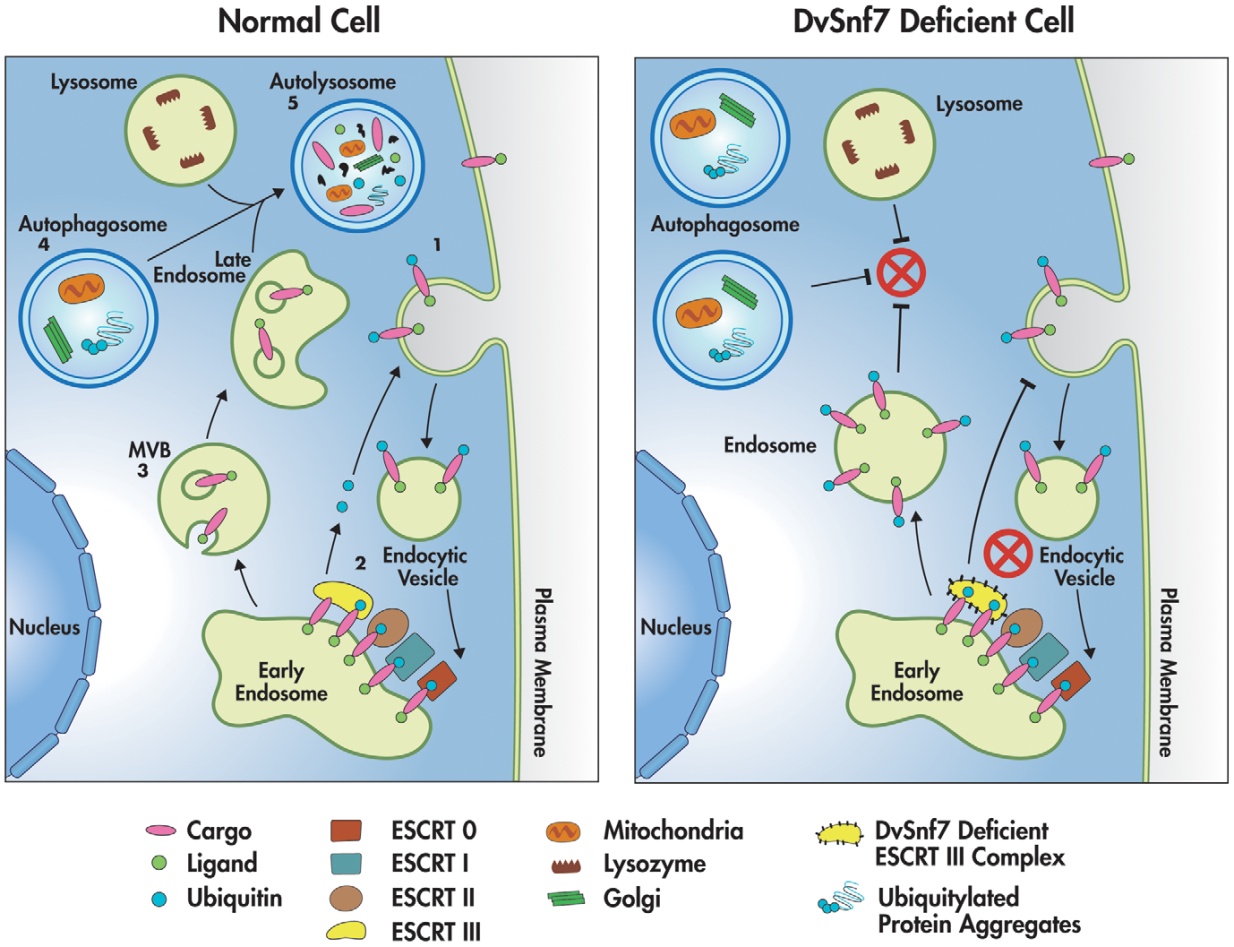
Model depicting endosomal-autophagy pathway involved in intracellular sorting and degradation of receptors along with other macromolecules in a normal cell (left) and a DvSnf7 deficient cell (right). In the normal cell, internalization and ubiquitination of cargo (1), de-ubiquitination of cargo (2), biogenesis of MVB (multi-vesicular bodies) (3), formation of autophagosomes engulfing macromolecules (4), and fusion of late endosomes, autophagosome and lysosomes into autolysosmes for degradation of cargo and macromolecules (5) are depicted. In the DvSnf7 deficient cell, the impairment of de-ubiquitination, accumulation of autophagosomes, and failure of fusion of endosomes, autophagosomes and lysosomes and autolysosmal activity are highlighted.

Based on the data collected by tracking the distinct steps of the endosomal-autophagy pathway at the cellular level in WCR larval tissues, we propose the following model (Fig. 5): in the normal cell, the endosomal-autophagic pathway plays a role in cellular homeostasis by internalization, transport, sorting and degradation of receptor cargo proteins [21]. Proteins that receive an ubiquitin tag are internalized into endocytic structures (1) which then fuse to the early endosome. In the endosome membrane, the ubiquitinated proteins are recognized by the ESCRT pathway (ESCRT-0, -I, -II, and –III complexes). The cargo proteins are de-ubiquitinated and the free ubiquitin is then recycled (2). The de-ubiquitinated proteins are sorted by budding off of endosome membrane leading to the formation of multivesicular bodies (MVB) (3). MVBs are late endosomes that accumulate small vesicles internally containing cargo proteins. Finally, late endosomes fuse with the lysosomes forming the autolysosomes where proteins are degraded. Concomitantly, intracellular organelles and long-lived proteins (ubiquitin aggregates) also undergo autophagic degradation. This process is amplified under starved conditions. These organellar components are engulfed in a closed double layered vacuole termed autophagosomes (4). The autophagosomes fuse either with the late endosomes or directly with the lysosomes forming autolysosomes (5), which leads to the final degradation of the incorporated materials.

In the DvSnf7 suppressed cells, endocytosis of ubiquitinated proteins appear to be functioning normally (as indicated by avidin incorporation in DvSnf7 dsRNA-fed insects in the present study). The ubiquitinated proteins are then sorted by the ESCRT pathway, however the de-ubiquitination of these proteins is impaired due to insufficient DvSNF7 in the ESCRT-III complex. The ubiquitinated proteins accumulate in the endosomal membrane (as evident in perinuclear localization of ubiquitin puncta in DvSnf7 dsRNA-fed larvae in the present study). Failure of the ESCRT-III assembly disrupts MVB formation [15], as demonstrated by the absence of MVBs in mouse neurons treated with mSnf7-2 siRNA [9] and to a lesser extent of MVB biogenesis in *Drosophila Vps32* mutant when compared to wild type [16]. Also, multiple lines of evidence suggest that autophagosomes accumulate in insect and mammalian cells as a result of Snf7 RNAi or Snf7 loss-of-function mutations [30], as well as in other ESCRT subunit mutants [14], [35], [43], [48]. Autophagy might be impaired by the absence of autolysosomal activity (as demonstrated by the absence of Lysotracker staining in DvSnf7 dsRNA-fed larvae, even under stimulated conditions in the present study). This might be due to a failure in the fusion of autophagosomes and endocytic vesicles to the lysosome compartments, as ESCRTs have been implicated in the fusion of autophagic vesicles and endocytic pathway for lysosomal degradation [14], [49]. In addition, Raiborg &, Stenmark [20] inferred that ESCRT is involved in final closure of the autophagosome, which is essential for the fusion with lysosomes and control of the number of autophagosomes. Hence, the apparent reduction in the Lysotracker staining in DvSnf7 RNAi insects could be an indirect effect as a result of failure of fusion of autophagosomes and endocytic vescicles to the lysosomes or final closure of the autopahgosome leading to impairment of autophagy.

In summary, suppression of DvSnf7 in WCR larvae leads to accumulation of the ubiquitinated proteins that are destined for lysosomal degradation. The sorting of transmembrane receptors through the endosomal pathway is a critical feature of eukaryotic cell biology, in part because of its ability to regulate signal transduction. Several cellular signaling pathways use ESCRT pathway for sorting and degradation of their membrane receptors such as Notch and epidermal growth factor [28], [29], [33], [46]. Moreover, suppression of DvSnf7 in WCR larvae also may lead to impairment of autophagy. Bulk degradation of damaged organelles and mis-folded cytoplasmic proteins by autophagy is also an important process for control of many physiological and pathological conditions in higher organisms [14], [15]. Taken together, these data suggest that DvSnf7 is the single WCR functional homolog of yeast Snf7 and its suppression, via oral delivery of DvSnf7 dsRNA in WCR larvae, impairs cell homeostasis and functioning in multiple tissues leading to mortality of WCR larvae.

## Materials and Methods

### Insect Rearing

WCR eggs from Crop Characteristics (Farmington, MN) were placed in a 25°C incubator for 13 days. WCR diet was prepared according to the recipe for Bio Serv SCR diet with a few adjustments, which included the addition of formalin at 0.06% per volume and corn root tissue (0.62% w/v), and the addition of 10% KOH to increase the pH to 9.0. Freshly hatched neonates were transferred to diet plates (96 well; Falcon) with 200 µl of diet per well. The plates were then incubated for 5 days at 27°C. Second instar larvae were used for the assays.

### Southern Blotting

A Southern blot was performed on gDNA of third instar WCR as previously described [50]. Briefly, DNA was extracted from larvae using the E.Z.N.A.® Insect DNA Kit (Omega Bio-Tek) according to manufacturer’s directions. Ten micrograms of gDNA was digested with HindIII (Fermentas), NsiI (new England Biolabs), or NdeI (Fermentas) for 18 hours, ethanol precipitated, resolved on a 0.8% agarose gel, and transferred to a positively charged nylon membrane (Hybond-XL, GE LifeSciences). The blot was probed with a ^32^P-labeled probe corresponding to exon 1 of the Snf7 gene using the RadPrime DNA Labeling System (Invitrogen). Hybridization was performed at 65°C in PerfectHyb Buffer (Sigma) and final washes of the blot were performed with 0.5×SSC, 0.1% SDS at 65°C. Blots were imaged on Kodak Biomax MS film.

### dsRNA Synthesis and Purification

The DvSnf7 target was amplified out of WCR neonate cDNA prepared using SuperScript™ First-Strand Synthesis System (Invitrogen) using total RNA extracted with TRIzol reagent (Invitrogen) following the manufacturers’ protocols. Primers [31] with a T7 polymerase promoter region (TAATACGACTCACTATAGGG) at 5′ end of the primers were used to amplify a DvSnf7-240 bp region by PCR. The PCR products were cloned into the pUC19 vector (New England Biolabs) between the EcoRI and HindIII restriction endonuclease recognition sites and were sequence confirmed. The DNA plasmids were linearized using a HindIII restriction enzyme and used as template for *in vitro* transcription using MEGAscript kit (Ambion) according to the manufacturer’s protocol.

The GFP sequence was cloned into pBTA2 vector and PCR amplified using gene specific primers [31] containing T7 polymerase promoter region at the 5′ end. The PCR product was used as template to make dsRNA with the MEGAscript kit (Ambion) following the manufacturer’s protocol.

### dsRNA Oral Feeding Bioassays and Preparation of WCR Tissues

The bioassays were performed using a diet-overlay method. A 20 µl aliquot of the dsRNAs (1 µg/ml of diet) was applied to the surface of the diet distributed in 96-well plates (one insect per well for a total of 8 insects per replicate and three replicated per treatment). The plates were allowed to air dry prior to adding one insect per well, and then incubated for 5 days at 27°C in the dark.

After 5 days of exposure to dsRNA, WCR larvae were removed from assay plates and briefly rinsed in 1×PBS (Phosphate Buffered Saline; Roche). The fat body and midgut tissues were dissected under a light microscope (Olympus) and used for the analysis.

### Real-time RT-PCR

RNA was extracted from insect tissues using TRIzol reagent (Invitrogen) following the manufacturers’ instructions. The RNA was then quantified using a spectrophotometer (Nanodrop), diluted to 50 ng in RNAse-free water, and used as template for real-time RT-PCR using the CFX manager software (Biorad), according to manufacturer’s instructions. The reaction included 1 µl of RNA (50 ng/µl), 6.25 µl of SYBR Green mix (*Iscript one-step RT-PCR* kit, BioRad), 0.5 µl of 10 pmol of forward and reverse primers [31], 0.25 µl of reverse transcriptase (BioRad) and 4.0 µl of RNAse free water, in a total volume of 12 µl. The reactions were set-up in 96-well format Microseal PCR plates (Biorad) in triplicates. Appropriate controls such as no-template control (NTC) and no reverse transcriptase control (NRT) were included. The endogenous control, tubulin, was used for normalization. A standard curve was generated using pUC-DvSnf7, a plasmid cDNA clone, to determine the relative RNA expression in larval samples.

### Endocytosis Tracking

Fat body and midgut tissues from five larvae per treatment were dissected 1×PBS and then transferred to a 1.5 ml tube containing 80 µg/ml of TR-Avidin (Invitrogen) in TC-100 insect medium (Sigma-Aldrich) with 5% bovine serum (Sigma-Aldrich ), 1X insect medium supplement (Sigma-Aldrich) and penicillin/streptomycin antibiotics (Invitrogen). The tissues were incubated for 15 minutes at room temperature with gentle agitation, rinsed two times and washed three times for 5 minutes with ice-cold PBS +0.5% BSA at 4°C. Tissues were then fixed in 4% paraformaldehyde (Electron Microscopy Sciences), washed three times for 5 minutes in 1×PBS and counterstained with DAPI (10 µg/ml; Sigma) for 5 minutes, and then mounted in SlowFade Antifade medium (Invitrogen). As negative control, a sub-set of samples was incubated in insect medium without TR-Avidin.

### Immunohistochemistry with Anti-ubiquitin Antibody

The fat bodies and midgut tissues were dissected as mentioned above and fixed in 4% paraformaldehyde overnight at 4°C. The tissues were washed three times in 1×PBS for 5 minutes each. Permeabilization of the tissues was performed with PBST (1×PBS +0.1% Tween-20) for 1 hour, changing solution every 15 minutes. Tissues were then incubated in PBSTG (1×PBS +0.1% Tween +5% Normal Goat Serum) overnight at 4°C. The tissues were exposed to the primary antibody (Anti-Ubiquitin antibody; Sigma) in PBSTG at 1∶100 dilution, overnight at 4°C. The tissues were washed in PBST (1×PBS +0.1% Tween-20) three times for 10 minutes each and incubated overnight at 4°C with secondary antibody (Alexa Fluor546, Roche, Goat anti-rabbit, Stock: 2 mg/ml) diluted 1∶1000 in PBST. The tissues were washed in PBST (1×PBS +0.1% Tween-20) three times for 10 minutes each, incubated in DAPI and mounted as described above. As negative control, a sub-set of samples was processed without the primary antibody (anti-ubiquitin).

### Autophagy Analysis

Fat body and midgut tissues were quickly dissected in 1×PBS under a stereo microscope and transferred to 50 µl dye solution containing 1 µM of Lysotracker Green DND-26 (Invitrogen) and 10 µg/ml of DAPI. The tissues were incubated in dye solution for 3 minutes at room temperature and then washed in 1×PBS two times for 1 minute. The tissues were mounted using 1×PBS and documented immediately under a confocal microscope. As negative control, a sub-set of samples was incubated in dye solution without Lysotracker Green DND-26.

### Apoptosis Assay

TUNEL assay was performed in fat body and midgut tissues using the In situ Cell Death detection kit (Roche), following the manufacturer’s instruction. As positive control, a sub-set of samples was treated with DNAse (Ambion). As negative control, the tissues were incubated in labeling solutions without enzyme.

### Imaging and Documentation

Imaging was conducted with Zeiss Laser Scanning Microscope (LSM) 510 META confocal microscope (Zeiss, Germany). Images were captured using a 488 nm (Lysotracker), 550 nm (Alexa-546, Texas Red), and 360 nm (DAPI) laser for excitation and 500–550 nm (Lysotracker ), 570 nm (Alexa-546, Texas Red) and 450–460 nm (DAPI) for the emission filter set, respectively. Fluorescent images were scanned simultaneously, and merged afterwards using LSM software (Carl Zeiss AIM version 4.2) for displaying of structural information. Figures of all micrographs were assembled using Adobe Photoshop CS5 software (version 12.0×32).

## Supporting Information

Figure S1 **Southern blot of WCR genomic DNA.** WCR gDNA was digested with HindIII, NsiI or NdeI and resolved on an agarose gel with a molecular weight marker (MW marker). The Southern blot was probed with a 32P-labeled probe corresponding to exon 1 of the Snf7 gene. Single bands for each restriction enzyme indicate that Snf7 is present in WCR as a single gene.
(TIF)

Figure S2 **Lysotracker staining of WCR tissues at higher magnification.** DsRNA for DvSnf7 (dsDvSnf7) and GFP (dsGFP) were overlaid on diet at 1 µg/ml of diet. The second instar WCR larvae were exposed to dsRNAs for four days and then starved for 24 h. Midgut and fat bodies were dissected from both sets of larvae and used for Lysotracker staining. Panels A–D show Lysotracker and nuclear staining (DAPI) merged images of starved samples. Green punctate marks denote Lysotracker staining that detects active acidic lysosomes undergoing autophagy (panels A &B). Scale Bar: 20 µm.
(TIF)

Figure S3 **TUNEL assays of WCR tissues.** DsRNA for DvSnf7 (dsDvSnf7) and GFP (dsGFP) were overlaid on diet at 1 µg/ml of diet. Second instar WCR larvae were exposed to dsRNAs for five days. An in-situ cell death detection kit was used for the assay. Panels A–D show FITC and nuclear staining (DAPI) merged images of midguts and fat bodies of both dsDvSnf7 and dsGFP treated insects. Panels E–H show FITC and nuclear staining (DAPI) merged images of midguts and fat bodies of both dsDvSnf7 and dsGFP fed insects that were treated with DNAse1 (positive control). Positive TUNEL staining (FITC) were observed in all panels (E–H). Scale Bar: 25 µm.
(TIF)
