# Supplementary figures and images for: Physiological and Cellular Responses Caused by RNAi- Mediated Suppression of Snf7 Orthologue in Western Corn Rootworm (Diabrotica virgifera virgifera) Larvae
Source: PLoS One. 2013 Jan 18;8(1):e54270. doi: 10.1371/journal.pone.0054270 (PMC3548817; doi:10.1371/journal.pone.0054270)

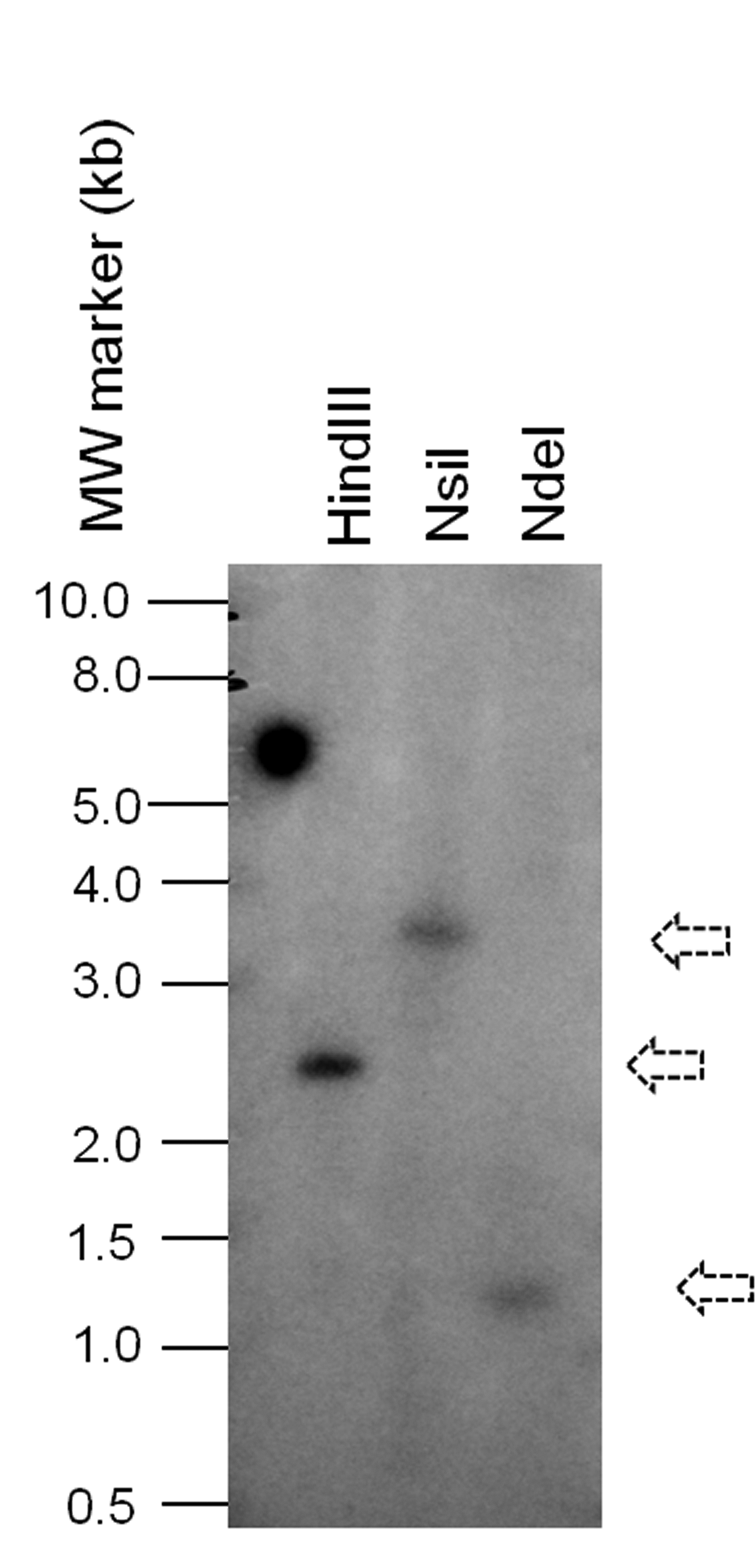

Supplement: Figure S1 — Southern blot of WCR genomic DNA. WCR gDNA was digested with HindIII, NsiI or NdeI and resolved on an agarose gel with a molecular weight marker (MW marker). The Southern blot was probed with a 32P-labeled probe corresponding to exon 1 of the Snf7 gene. Single bands for each restriction enzyme indicate that Snf7 is present in WCR as a single gene. (TIF) [file pone.0054270.s001.tif]

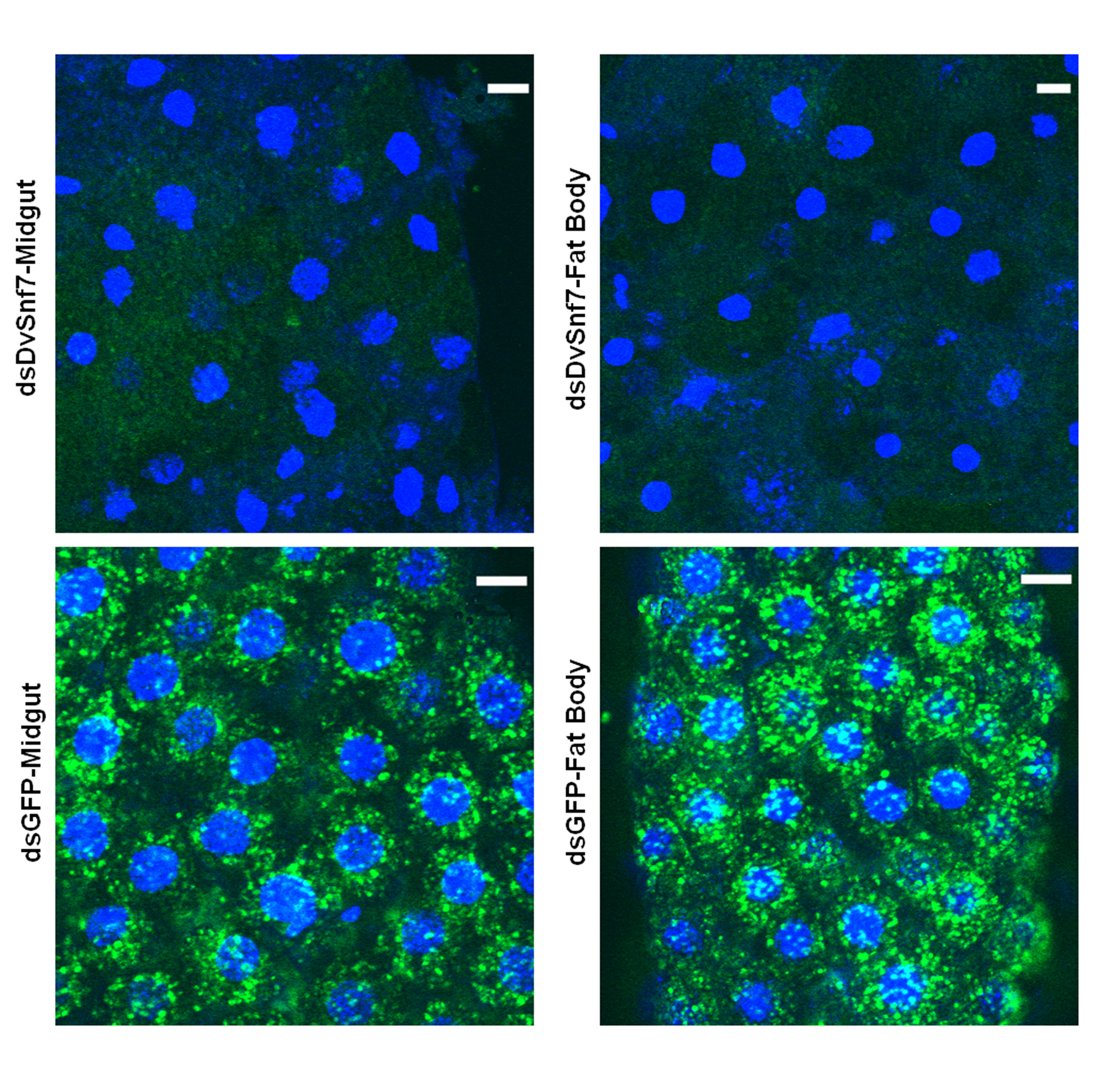

Supplement: Figure S2 — Lysotracker staining of WCR tissues at higher magnification. DsRNA for DvSnf7 (dsDvSnf7) and GFP (dsGFP) were overlaid on diet at 1 µg/ml of diet. The second instar WCR larvae were exposed to dsRNAs for four days and then starved for 24 h. Midgut and fat bodies were dissected from both sets of larvae and used for Lysotracker staining. Panels A–D show Lysotracker and nuclear staining (DAPI) merged images of starved samples. Green punctate marks denote Lysotracker staining that detects active acidic lysosomes undergoing autophagy (panels A &B). Scale Bar: 20 µm. (TIF) [file pone.0054270.s002.tif]

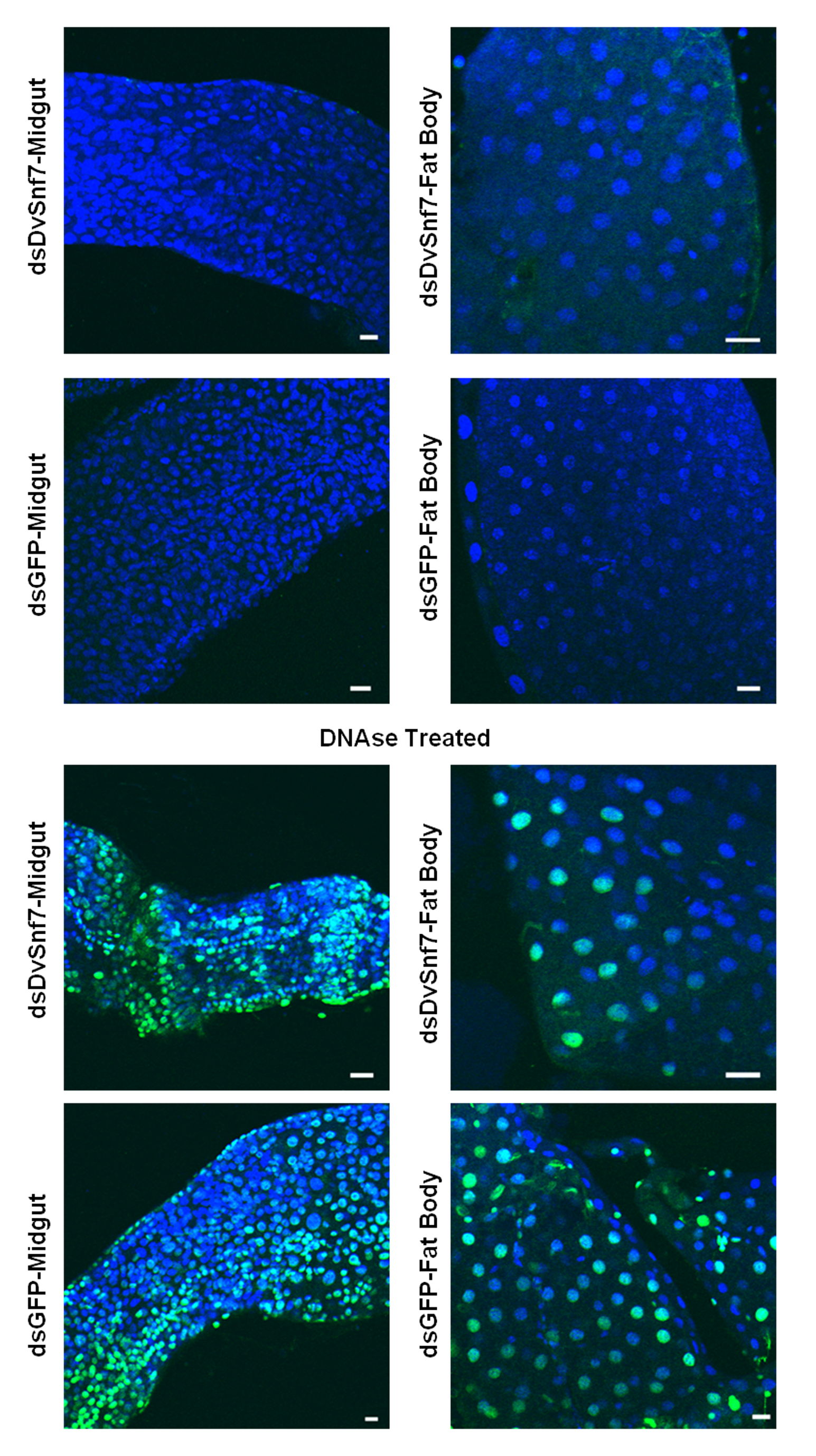

Supplement: Figure S3 — TUNEL assays of WCR tissues. DsRNA for DvSnf7 (dsDvSnf7) and GFP (dsGFP) were overlaid on diet at 1 µg/ml of diet. Second instar WCR larvae were exposed to dsRNAs for five days. An in-situ cell death detection kit was used for the assay. Panels A–D show FITC and nuclear staining (DAPI) merged images of midguts and fat bodies of both dsDvSnf7 and dsGFP treated insects. Panels E–H show FITC and nuclear staining (DAPI) merged images of midguts and fat bodies of both dsDvSnf7 and dsGFP fed insects that were treated with DNAse1 (positive control). Positive TUNEL staining (FITC) were observed in all panels (E–H). Scale Bar: 25 µm. (TIF) [file pone.0054270.s003.tif]
